# 1279. Vertebral Osteomyelitis due to *Serratia* spp: A Rare Entity Emerges as an Infection Linked to Injection Drug Use

**DOI:** 10.1093/ofid/ofad500.1118

**Published:** 2023-11-27

**Authors:** Briana Castillo, L Madeline McCrary, Asher J Schranz

**Affiliations:** University of North Carolina - Chapel Hill, Durham, North Carolina; Washington University in St. Louis, Chapel Hill, North Carolina; University of North Carolina, Chapel Hill, NC

## Abstract

**Background:**

*Serratia* spp. are environmental gram-negative bacilli known to cause invasive infection in the setting of injection drug use (IDU), but uncommonly associated with orthopedic infections. In recent years, in the setting of rising IDU, there have been mounting reports of bacteremia and endocarditis due to *Serratia* spp. Vertebral osteomyelitis (VO), another condition associated with IDU, has rarely been reported due to *Serratia* spp, with the largest published series reporting three cases. Given the rise of invasive *Serratia* infections and IDU, we characterized experience caring for VO due to *Serratia* spp. (S-VO).

**Methods:**

This was a retrospective review of adults with S-VO admitted to UNC Health, a hospital network in North Carolina, from 4/2014 to 6/2022. We utilized EMERSE, a program that searches free text in the electronic medical record, to identify charts with the terms ‘Serratia’ and ‘vertebral osteomyelitis.’ We identified 31 patients and included 10 in the final analysis (Fig 1). Chart review was performed to confirm the presence of S-VO and to obtain demographic and clinical data.

**Figure 1**

**Figure:**
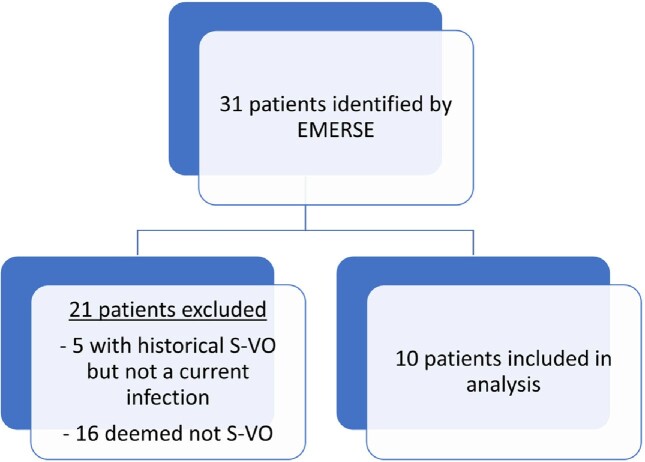

**Results:**

Cases overall increased over time; there were 0-1 per year until 6/2019, but 2 per year since (Fig 2). The median age was 45 years and 90% were White (Table 1). IDU was the main risk factor (8 of the 9 with risk factor information). There was a predilection for the thoracic spine (70%) and 50% were associated with epidural or paravertebral abscess. Although half had concomitant bacteremia, only one had concurrent endocarditis. The predominant species was *S. marcescens* (8 of the 9 with species-level identification), and the majority were monomicrobial. Most received ceftriaxone or fluoroquinolone monotherapy, and 30% underwent surgical management (Table 2). There were two confirmed failures or relapses and one death at 90 days after discharge.

**Figure 2**

**Figure:**
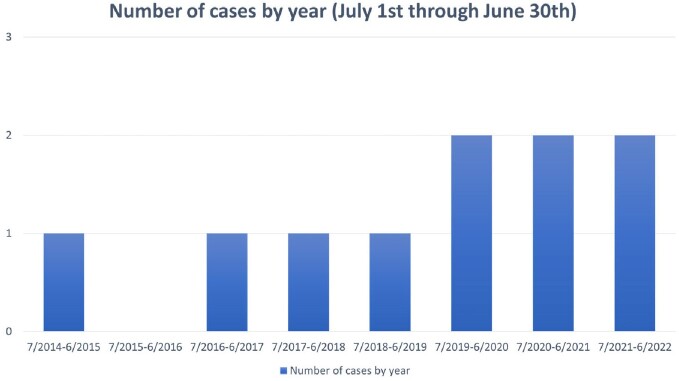

**Figure:**
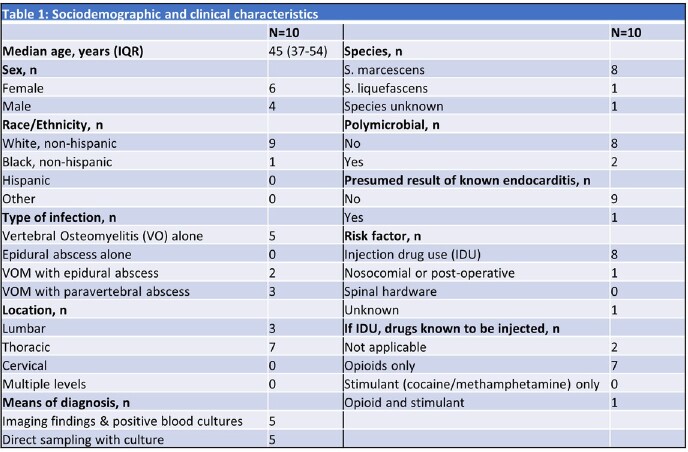

**Figure:**
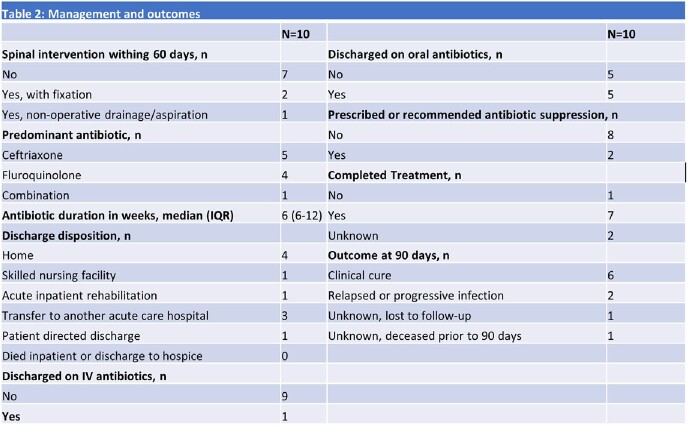

**Conclusion:**

Although considered a rare entity, we identified ten cases of vertebral osteomyelitis due to *Serratia* spp. within one health system over eight years. S-VO was largely linked with IDU, typically occurred in the absence of endocarditis and was frequently managed with a third-generation cephalosporin or fluoroquinolone. Further multicenter data is needed to define trends and elucidate management strategies for orthopedic infections due to *Serratia* spp.

**Disclosures:**

**Asher J. Schranz, MD, MPH**, WoltersKluwer: Honoraria

